# Group experiences of cognitive stimulation therapy (CST) in Tanzania: a qualitative study

**DOI:** 10.1080/13607863.2021.1872489

**Published:** 2021-01-18

**Authors:** Jasmine Morrish, Richard Walker, Catherine Dotchin, Aimee Spector, Stavros Orfanos, Sarah Mkenda, Esther Peniel Shali

**Affiliations:** a Population & Health Sciences Institute, Newcastle University, UK; b Northumbria Healthcare NHS Foundation Trust, North Shields, UK; c Division of Psychology & Language Sciences, University College, London, UK; d Kilimanjaro Christian Medical College, Moshi, Tanzania; e Hai District Hospital, Bomangombe, Hai District, Kilimanjaro Region, Tanzania

**Keywords:** Cognitive stimulation, Group Therapy, Tanzania, Dementia and Cognitive Disorders, Low-resource Mental Health Interventions

## Abstract

**Background:**

Tanzania is a low-income country in which medication for dementia is largely unavailable. Cognitive Stimulation Therapy (CST) is a group-based psychological treatment for people with dementia (PwD), shown to improve cognition and quality of life (QoL). It has previously been culturally adapted and piloted in Tanzania, shown to produce similar outcomes. UK research into CST suggests processes inherent to the group nature are key to its success. This study sought to identify group processes within CST in Tanzania and understand their impact on CST principles and outcomes.

**Methods:**

Data collection took place in rural Hai District, through qualitative semi-structured interviews. Sixteen PwD and four facilitators were recruited through convenience sampling and interviewed about their experiences of CST. Interviews were audio-recorded, translated, transcribed and analysed by thematic analysis.

**Results:**

Two main themes emerged: ‘Positive group experiences’ and ‘Negative group experiences’. From this, a number of group processes were identified, such as helping behaviours and feeling understood by the group. Positive processes supported CST principles and participant improvement. Facilitators were influential over group dynamics. The group processes identified impacted CST principles and treatment outcomes.

**Conclusions:**

This is the first study on group mechanisms of CST in Tanzania. It provides deeper insight into participants’ experiences of CST, thus identifying specific processes underlying the quantitatively measured positive outcomes of CST in Tanzania by previous studies. It also reveals further cultural barriers to implementation, enabling amendments for optimization of treatment efficacy.

## Introduction

The global burden of dementia is rapidly increasing. This is largely attributable to increased cases in low- and middle- income countries (LMICs) as the populations undergo demographic transition, with result in increase in the older population (Prince et al., 2008). Some of the largest increases are expected across sub-Saharan Africa (SSA) (Prince et al., 2015). A lack of geriatricians, neurologists and psychiatrists in low-resource settings is a major challenge for diagnosis and management of dementia; rendering pharmacological management inviable (Dotchin, Akinyemi, Gray, & Walker, 2012).

Cognitive stimulation therapy (CST) is a non-pharmacological, evidence-based intervention for dementia (Spector et al., 2003), shown to significantly improve patients’ cognition and quality of life (QoL) (Lobbia et al., 2019). CST is cost-effective (Knapp et al., 2006) and the only non-pharmacological intervention recommended by the UK National Institute of Clinical Excellence (NICE) to promote cognition, independence and wellbeing in mild-moderate dementia (NICE, 2018). CST improves cognitive and social functioning, through a broad range of activities (Woods, Aguirre, Spector, & Orrell, 2012). Groups are facilitated by a trained therapist, nurse or carer. Key principles include ‘maximising potential’ and ‘opinions rather than facts’; focussing on individual strengths (Spector, Gardner, & Orrell, 2011).

CST has been successfully adapted for use in SSA (Mkenda et al., 2016). Key issues addressed included working around market hours and provision of an additional facilitator to assist participants with sensory impairment (Mkenda et al., 2016). A feasibility study in Hai, Tanzania showed CST significantly improved patients’ cognition, QoL, anxiety and behavioural symptoms (Paddick et al., 2017). A Nigerian feasibility showed similar therapeutic benefits (Olakehinde et al., 2018). Practically and economically CST is suitable for use in low-resource countries as it can be delivered by non-specialist health workers, requiring little specialist equipment (Mkenda et al., 2016).

A recent RCT concluded Individual CST (iCST), delivered one-to-one by a caregiver, did not improve participants’ cognition or QoL (Orrell et al., 2017), suggesting different mechanisms may occur within each format. A UK qualitative study by Orfanos, Gibbor, Carr, and Spector (2020) identified group processes within CST, including ‘importance of companionship’ and ‘cognitive stimulation through group interactions’. Challenges included expressing one’s views, with authors suggesting overcoming challenges aid self-development. Other research suggests groups facilitate social interaction, through which self-expression develops (Lobbia et al., 2019).

A qualitative study showed factors fundamental to the group format were linked to positive experiences, highlighting conversational aspects and a supportive environment (Spector et al., 2011). In a Brazilian adaptation study, participant bonding improved attendance, and the group format enabled helping behaviours, facilitating CST (Bertrand et al., 2019). An adaptation study in Hong Kong found, although feasible, adaptations were required to overcome cultural barriers to participation and interaction (Wong et al., 2017). In the UK, a shared dementia diagnosis made PwD feel comfortable and less worried about embarrassing themselves (Bailey, Kingston, Alford, Taylor, & Tolhurst, 2017). More generally, group psychotherapy is recommended in older people to alleviate loneliness (Cattan, White, Bond, & Learmouth, 2005).

To the authors’ knowledge, no previous studies have qualitatively explored group mechanisms behind CST in SSA. This may help further identify therapeutic advantages and/or disadvantages of the group format, which could subsequently be reinforced or reduced to enhance therapeutic effect. This study aimed to gain insight into experiences of group processes and their impact on CST principles and outcomes, through exploring perspectives and experiences of participants and facilitators of Tanzanian CST.

## Methods

### Setting

Data collection was undertaken in four villages in Hai District, in the Kilimanjaro region of northern Tanzania. Most inhabitants are subsistence farmers; others produce cash crops including tomatoes and coffee (Paddick et al., 2017). The main language spoken is Swahili, and the Chagga are the largest tribe. In older adults, educational attainment is low- over two-thirds of older women received no formal education (Longdon et al., 2013). Each village has an enumerator, previously involved in healthcare research collecting census data (Setel, Kitange, Alberti & Moshiro, 1998).

### Participants

#### People with dementia

The methodology from a recently published UK study exploring CST group processes (Orfanos et al., 2020) was used for this present study, enabling cross-cultural comparison. 15-20 PwD was the intended sample.

For PwD inclusion criteria was as follows: (a) Currently attending or had attended CST groups within the last 10 weeks, (b) DSM-IV criteria for mild to moderate dementia confirmed by consultant psychiatrist or geriatrician, (c) had sufficient memory of groups as determined by the researchers, (d) had sufficient verbal communication to conduct interview, (e) could provide full verbal and written consent. The demographics of PwD are summarised in Table 1. 

**Table 1.** Table summarizing PwD demographics.

Characteristic	**PwD (** *n* ** = 16)**
**Gender**	
Male	6
Female	10
**Age**	
70-79	4
80-89	8
90-99	3
≥100	1
Mean age in years (standard deviation)	82.3 (7.3)
**Religion**	
Christian	15
Muslim	1
**Tribe**	
Chagga	14
Massai	1
Pare	1
**Educational Level**	
No formal education	15
≥8 years formal education	1

#### Facilitators

Inclusion criteria for facilitators was that they had experience leading at least one recent CST group in Hai. Of the four facilitators interviewed, one was a senior and three were recently qualified occupational Therapists (OTs), having each received the standard one-day CST training course.

### Procedure

#### Interviews

Semi-structured interview topic guides were developed for the UK study, one for PwD; another for facilitators (Orfanos et al., 2020). These were adapted for use in Tanzania and translated into Swahili. Throughout data collection, the topic guide was revised and modified according to post-interview feedback from the interpreter. The topic guides (see Appendix 1) provided a flexible structure, following three main points:

- General experiences of being in the group
- How group experiences impacted CST principles
- Whether they felt group experiences impacted their cognition/QoL

Interviews were undertaken in in English; interpreted into Swahili by an experienced local translator (ES). A relative, referred to as the caregiver, translated tribal languages not spoken by the interpreter into Swahili. They were asked to translate the exact words spoken by the PwD and interviewer.

Interviews were recorded using recorded on a Sony ICD-PX370 Dictaphone. Contemporaneous field notes were taken, including observations of body language.

Interviews with PwD were undertaken in their homes or at the local dispensary, all with a caregiver present. Interviews with PwD lasted between 19 and 57 min (mean= 36). A bag of sugar was deemed an appropriate gift to thank participants. Facilitator interviews took place at the regional hospital, Kilimanjaro Christian Medical Centre (KCMC). Facilitator interviews lasted between 46 and 70 min (mean= 60).

#### Translation and transcription process

Translation and transcription of interview recordings from Swahili to English were undertaken by three experienced local translators. The researcher and translator reviewed each transcript together, ensuring accurate interpretation of meaning. The translators were unable to transcribe certain dialects; in these cases, the caregiver’s Swahili translation was used in the results.

#### Ethical approval and consent

The study received ethical approval locally from KCMC Research Ethics Committee and nationally from the National Institute for Medical Research, Tanzania.

Written consent was obtained by signature or thumbprint. Participants who met the inclusion criteria were expected to have capacity to consent themselves for participation, however time was taken to allow participants to decide. Verbal and written information was given to all participants in their preferred language and read aloud to illiterate participants.

### Data analysis

Transcripts were analysed by thematic analysis; stored and coded on NVivo coding software. Braun and Clarke’s guidance (2006) was followed over a five-stage process: (1) familiarisation of transcripts, (2) generating recurrent data into codes, (3) collating codes into themes, (4) reviewing of themes and (5) refinement of themes. Aligning with guidance, the frequencies of codes within themes were not included as the research intends to provide deep description of social phenomena rather than quantify empirical material (Ritchie, Lewis, Lewis, Nicholls, & Ormston, 2013). Furthermore, considering the small sample size, frequencies hold little significance and may be misleading (Ritchie et al., 2013). To increase trustworthiness, inter-rater credibility checks of codes and themes were undertaken by external qualitative researchers. An inductive approach was used in analysis with data-driven codes and themes (Silverman, 2010). The study adopts a phenomenological approach, in which the researcher seeks to understand a phenomenon by exploring it from the perspective of individuals who have experienced it (Teherani, Martimianakis, Stenfors-Hayes, Wadhwa, & Varpio, 2015).

## Results

### Sample

21 PwD were recruited through enumerators from four recent CST groups across Hai district. Of these, two had no memory of CST and three did not have a formal dementia diagnosis. When screened for cognitive impairment using the IDEA cognitive screen (Gray et al., 2016; Paddick et al., 2015), they scored in the lowest group and were selected for and attended CST. When assessed by a psychiatrist, they did not meet DSM-IV criteria.

### Themes

**Figure 1.**
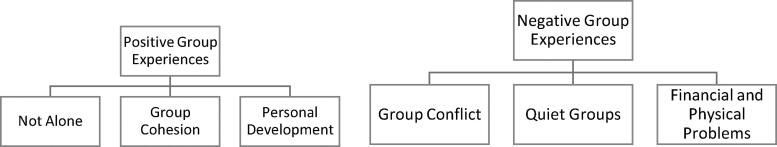
Summary of themes and subthemes.

This study sought to explore participants’ and facilitators’ group experiences of Tanzanian CST. Thematic analysis revealed 2 main themes, each containing 3 subthemes, summarised in figure 1:

### Theme 1—Positive group experiences

#### Subtheme—Not alone

Feelings of social isolation were common among participants, often due to poor physical health. They could not work or visit friends so stayed alone at home. Attending groups alleviated loneliness and participants felt more active during the 7 weeks of CST.

*‘Whilst being at the group I was more active. I walked to the group, now I am able to walk slowly to go visit my neighbours.’ – Participant 12 (P12)*

Being around others provided distraction from physical illness. When the sessions finished, participants became lonely and felt they deteriorated. Other participants reported an increase in physical activity, walking to visit nearby friends.

*‘It (being in the group) helped her a lot because she was very happy, she was very active when she came from the group and her memory was so active, but when the groups ended, she started to be sick again. She is not active again and felt depressed.’ – Caregiver of P12 (C12)*

PwD felt similar to other members. Prior to CST, many were unaware other people experienced memory problems, but in groups they felt understood.

*‘No, I didn’t tell anyone (about my memory problems) because everyone has memory problems and everyone at some point says, “I don’t remember this”… Yes, we laughed, one woman says I don’t remember this, then others can say “I even don’t remember!”’- P13*

Being around other people of a similar age put participants at ease and enabled them to share memories. Some knew each other from childhood so reminisced together. Talk of the past was used to initiate discussions on current topics.

*‘She went there and met with other women, they were the same age, they sang the songs they used to sing in the past in circumcision celebrations. They asked her “can you sing the songs for us?” and she sang the song and other women sang back up.’ – C16*

*‘They talked a lot about the past… They were able to recall a lot of things they used to do when they were young and compare with current things.’ – Facilitator 1 (F1)*

#### Subtheme—Group cohesion

The groups were described as a safe space. Participants trusted other members over people outside the group. This was supported by facilitator observations.

*‘If I mix with people from inside the group my memory will continue to improve but mixing with other people with memory problems outside the group might make my memory worse because they don’t have the same skills we gained…’ – P1*

*‘It seems like there are things she could not say to anyone else, but she was kind of free to talk in the group.’– F1*

Overall, participants enjoyed attending groups, expressing positive feelings for other members. They felt respected, included and a sense of belonging. Laughter, particularly during activities, was often reported.

*‘I think they felt like they belong somewhere… for them having that badge and sitting in the group, having the group name and the song made them feel more part of the group.’– F4*

*‘We laughed, we were doing different activities. We made mats, pot mats and we danced the traditional songs. We played like kids.’ – P13*

Participants worked together, with those more able helping and encouraging others. They reminded each other of past memories. Many led group activities, encouraged by facilitators. Participants physically assisted others, helping those less able to walk to sessions, rather than taking transport.

*‘Each one knew to make different things, so they (the other group members) helped her to hold some material and she made a pot mat.’- C16 (P16 visually impaired)*

*‘There was this one who couldn’t see, in the first times we used to fetch them (by car), but there were other ones who could just walk her home, and she was like “okay, I won’t take a car, I just walk home.”’ – F1*

In rural Tanzania, although the majority speak Swahili, many older people speak only their tribal language. Facilitators could not speak local dialects. As participants lived in close proximity, many spoke the same dialect. Those who spoke both translated for others.

*‘There was someone who could understand Swahili and translate it to their language. That means the group work together more closely. So, when they were interacting in their local language, you could see the participation was higher.’ – F4*

#### Subtheme—Personal development

Isolated participants lacked interaction at home- families automatically attended their needs. Groups were a safe space to practice social interaction. Participants and facilitators saw improvements in communication skills, behaviour and self-control. PwD reported feeling mentally stimulated after attending groups.

*‘Sure, there are changes, very big changes. The way I used to speak in an uncontrolled way, shouting abuse, being rude… and now I have more control over what I say, where I was not listening well- for sure it has helped.’ – P1*

*‘At the beginning… they were not communicating, came silent. But as time goes on, we find them trying to express words, trying to socialize with each other.’ – F3*

Attending the groups structured participants’ weeks, giving them something to look forward to. On the morning of the group, participants prepared themselves early. Some started walking to groups themselves.

*‘The changes are as if he remembers today that the car is coming, he prepares himself, showering well, dressing well, he has changed.’ – C6*

Through activities, participants were reminded of previous household roles. There was an increase in home activities undertaken. The programme also increased understanding and acceptance of dementia by the wider community.

*‘I’ve started to do more of my home activities slowly, one by one.’ – P1*

*‘She was happy before; she was talking about the group every time with her grandsons and they laughed about it, saying she was ‘graduating’ from school.’ – C12*

### Theme 2—Negative group experiences

#### Subtheme—Group conflict

Individuals’ challenging behaviour upset others and caused distraction. Certain members pointed out others’ incorrect answers. Whilst some dominated conversation, others required persuasion to participate in activities that they initially felt were too childish.

*‘…one of the members dominates others, all the time speaking. And if another wants to speak, they say "stop! You don't know about that.”’ – F3*

Differences in religious beliefs caused friction and limited conversation. Facilitators intervened if tensions arose. Each session was opened with a group song. A church song was once chosen, which upset individuals of other beliefs.

*‘It can affect the socialization because many people… discuss religion, if not religious it’s politics, if not politics it’s gossips. If you cannot discuss politics because you don’t read newspapers or you don’t watch television, you don’t have much to talk about. So the only thing many people talk much, here is religion. We sit in a group and you are feeling ‘I can’t talk frankly because of the Muslim people’ or Muslim people think ‘I don’t want to hear what Christians are going to say about me.”’ – F4*

One group mostly contained Maasai Tribe members, in which men are deemed more powerful than women. The facilitator noticed another predominantly female group spoke more openly. Many reported a gap was left between chairs. In one group, men and women sat separately.

*‘Sometimes there is this issue of gender balance, maybe in the communities, men are more powerful than women… They tell other people to be quiet “because I am a man”. So, it is also observed that in the Maasai area… “I am speaking, why are you speaking? Wait until I finish.’ – F3*

*‘He said “when they were together with my wife here, if there is anything I want, I want something, I tell her speak, do this and that.”’ – Wife of P6*

Participants of higher social status were recommended for leadership more often. Those from lower educational or financial backgrounds were less trusted. Illiteracy was common; some struggled to understand activities, reducing participation. Intertribal differences existed- the Maasai tribe generally had less formal education.

*‘I find someone saying “ah… this person doesn’t know anything… because, (they’re) not able even to go to school.”… By seeing the person maybe has worn clothes and the way he is speaking… also can bring the poor trust to others.’ – F3*

Living within small communities, participants already knew each other; some families had unresolved conflicts, for example over land. Facilitators were mindful of this when leading discussions. Some avoided sharing personal problems due to fears of gossip.

*‘If I quarrelled with my husband or my son, why would I tell anyone?… Because there are people who gossip a lot.’ – P9*

*‘She didn’t tell them her secrets, like ‘I slept without having eaten’ or ‘I woke up without eating anything.”’ – C12*

#### Subtheme—Quiet groups

Some groups were less talkative than others. Facilitators encouraged involvement; however, relatives were asked to attend particularly quiet groups. Active participation was lower in afternoon groups than the morning due to tiredness.

*‘It was challenging because it was done in that group, which was not so active, it didn’t have an active person to get it going unless the facilitator would prompt people.’ – F1*

Groups were run in Swahili. Members who spoke only their tribal language found it difficult to contribute, despite bilingual individuals translating. Facilitators struggled to navigate cultural intricacies. When participants spoke in their local dialect, interaction increased.

*‘We facilitators were not familiar with things like language… and also their culture… I am a Chagga from Kilimanjaro, but I come from Marangu not Masama… they speak very different local languages, their culture is also different… So, sometimes it makes it difficult to bring in… examples which relate to their culture.’ – F2*

Although many PwD said it was easy to speak in groups, facilitators noticed concerns over answering incorrectly. When participants did not understand, they remained quiet. Although members were reassured there were no wrong answers, these were often laughed at. Participants waited for instructions, even when given a choice over activities. Facilitators felt this may be linked to a perceived paternalistic healthcare professional-participant relationship.

*‘There are some activities maybe they find difficult to do… for those ones who have never been to school they are afraid it is like a test, we are testing their intelligence.’ – F4*

#### Subtheme—Financial and physical problems

Physical abilities varied; those with poor health sometimes struggled to participate, becoming discouraged and feeling they gained less from sessions. Facilitators used alternative, time-consuming communication strategies to include those with sensory impairments, but other members became impatient.

*‘One thing which made her feel bad is where others can stand up and dance, but she was not able to do that due to her weakness.’ – C12*

*‘In the group she felt like her memory was worse than others because other people were more active than her, so she felt a lot of pressure.’ – C15*

Talk of physical illness distracted participants. Many complained, expecting drug treatments. Facilitators often had to re-explain the purpose of CST and redirect the focus to activities.

*‘They would think that even they met there for their other physical problems not even for dementia, so we had to remind them over and over because they'll be like “Ooh my eye really hurts, ooh my blood pressure is very high.” So, we have to keep on telling them “now this group is for memory… Because they kept on pushing like “we really want medication.”’ – F1*

Financial hardship was common. Participants asked facilitators for financial help, inciting others to ask. Some struggled to afford food, affecting their physical health and concentration. Participants were given a fizzy drink and donut at the end of each session, which for many was important.

*‘There were some elderly who were telling us that “you know I am not only having memory problems”…“This problem at home, I don’t have food.”…They were thinking that we can offer them money to cover some problems they were facing.’ – F2*

Financial difficulties and physical illness were barriers to socialising outside groups. Although they formed friendships and noticed improvements in themselves, most returned to feeling isolated and inactive after the last session. Participants expressed wishes for the groups to continue and were sad they had finished.

*‘We were telling them “you know after this session the next week will be our last session,” they were complaining: “So after next week, how are we going to meet again?”…it was really so frustrating to them.’ – F3*

*‘When she was with other women in the group, she was very happy… For a short time.’ – C15*

## Discussion

To the authors knowledge, this is the first study to qualitatively explore group mechanisms in Tanzanian CST. Two overarching themes arose: ‘Positive Group Experiences’ containing subthemes ‘Not Alone’, ‘Group Cohesion’ and ‘Personal Development’ and ‘Negative Group Experiences’ containing ‘Group Conflict’, ‘Quiet Groups’ and ‘Financial and Physical Problems’.

### Benefits of the group format in Tanzanian CST

The opportunity for social interaction is a well-recognised benefit of group CST (Bailey et al., 2017; Bertrand et al., 2019; Dickinson, Gibson, Gotts, Stobbart, & Robinson, 2017; Orfanos et al., 2020). Through this, aligning with Swaab’s “use it or lose it” hypothesis, participants can redevelop “present, yet under-rehearsed cognitive skills” (Swaab, 1991). PwD and facilitators noticed increased socialisation attempts and improvements in conversational abilities, similar to findings of Spector et al. (2011), fulfilling CST principles ‘stimulating language’ and ‘building and strengthening relationships’. Meeting similar people helped participants feel understood and normalised forgetfulness, supporting previous qualitative research (Bailey et al., 2017; Orfanos et al., 2020; Spector et al., 2011).

Being of similar ages, participants shared past experiences and discussed previous household roles. Creative sessions, such as making pot mats or singing/dancing to traditional music, as well as the sessions on childhood, using money, food and orientation elicited conversations about the past. Facilitators described how this led to present day comparisons, facilitating CST principle ‘using reminiscence as an aid to the here-and-now’.

Overall, good cohesion was described, mirroring existing studies (Orfanos et al., 2020; Spector et al., 2011; Wong et al., 2017). Participants felt trusted and accepted. Both of these positively correlate with group cohesiveness (Roark & Sharah, 1989), thought to be strongly related to patient improvement (Burlingame, Fuhriman, & Johnson, 2001). Principles ‘Inclusion’ and ‘building/strengthening relationships’ are facilitated by good cohesion and a sense of belonging. The group song which opens each session helped achieve the above experiences.

In common with Orfanos et al. (2020) was enjoyment of being with the group. Participants particularly enjoyed creative sessions, in which they described laughing and playing like children, aligning with the principle ‘fun’. Similarly, PwD in Hong Kong engaged better in activities involving actions, possibly resulting from the ‘tangible’ success they offer or cultural preference for deeds over words (Wong et al., 2017).

In this present study, many helping behaviours were identified. In Brazil, these helped explain how the group format facilitates CST (Bertrand et al., 2019). Mason, Clare, and Pistrang (2005) found self-esteem improved through helping others. ‘Group support’ is a theme identified by Orfanos et al. (2020)- helping others and feeling helped by the group. In this present study, helping behaviours facilitated inclusion of less able members and encouraged new initiatives to walk to groups. Participants felt more active after groups, undertaking more home activities and personal care, reflecting findings of Spector et al. (2011). These findings support those of the Tanzanian feasibility study where significant improvements were seen in the physical health domain of QoL (Paddick et al., 2017). Woods, Thorgrimsen, Spector, Royan, and Orrell (2006) suggest improvements in physical activity improve PwD’s QoL

PwD reported improvements across many other domains including feeling mentally stimulated, improvements in cognition, conversational skills, and behaviour/self-control. This supports findings of Paddick et al. (2017), in which significant improvements in all three domains of the ADAS-Cog (language, memory/new learning, and praxis) were shown.

Improvements in behaviour and self-control can be explained by group norms. Over time normative social influence occurs- participants comply with group norms to gain acceptance (Kelman, 2005). They eventually internalize group values, such as listening to and respecting others; group control develops into self-control (Kelman, 2005).

One facilitator described how in Tanzanian culture; relatives undertake household activities for older people out of respect. This is an example of unintentionally produced “malignant social psychology”- the interaction style resulting in devaluation and loss of personhood (Kitwood, 2002). Groups counteracted this, providing a forum for participants to express ideas and opinions and complete tasks themselves.

### Interconnecting nature of group processes in CST

Orfanos et al. consider an inter-connection between group processes (Orfanos et al., 2020). The present study supports this, showing that from certain experiences (i.e. feeling similar) a number of others arise (feeling comfortable to speak, interaction with others, sharing memories, good cohesion). Although similar group processes are likely to exist in other psychosocial group interventions for dementia, how they are elicited in CST is unique to the structure of sessions and themed activities. Each activity draws out multiple interconnecting group processes, enhanced by weekly variation. Continuity and consistency are achieved through weekly repetition of certain activities such as the group song and general structure of sessions.

### New insights into challenges of Tanzanian group CST

Challenges arose from previous conflicts (i.e. over land ownership) and fears of gossip. Conversely, in the adaptation study, caution was required to avoid oversharing personal information (Mkenda et al., 2016). The facilitator is key in directing conversations appropriately. Living in close proximity also provided benefits such as walking home together and collaborative memories of past village events. Facilitators attributed the need for prompting and fears of answering incorrectly to the paternalistic healthcare provider-patient relationship observed in Tanzania. This was potentially exacerbated by the facilitator-participant language barrier, as interaction increased when speaking local dialects. In Hong Kong, participants seldom expressed opinions due to a cultural ‘cautiousness/conservatism’ and for fear of disrupting group harmony (Wong et al., 2017). In UK studies, PwD interacted and engaged unprompted (Spector et al., 2011) and disclosed personal feelings within groups (Mason et al., 2005; Orfanos et al., 2020).

Orfanos et al. (2020) suggest challenges of the group format may act as a “catalyst for learning and therapeutic change”. For instance, the facilitator-participant language barrier seemed to enable helping behaviours. Forsyth (2010) suggests recognition of the leader’s authority can be beneficial as it increases compliance with therapeutic directives.

In Tanzanian CST, tribal and religious differences produced more conflict than in other countries. Culturally, these significantly contribute to one’s identity. The Tanzanian adaptation study highlighted a need to avoid holding sessions in buildings of religious worship to avoid alienation of faiths (Paddick et al., 2017). Participants engaged well with church songs (Mkenda et al., 2016), however the present study showed religious differences limited the scope and depth of conversations. Tribal differences resulted in differing educational backgrounds and views on gender roles. Wong et al. (2017) suggest the ‘slightly different yet compatible cultures’ may facilitate discussion, despite differences in dialects posing communication barriers.

Other influencers of group experiences were participants’ adverse financial situations and physical health. These issues caused distraction and expectations of treatment were unmet. Most cases of non-completion in both Tanzania and Nigeria were due to these unmet expectations and failure to understand non-pharmacological treatment (Mkenda et al., 2016). In Nigeria, participants’ blood pressure was taken with appropriate referral by nursing staff to help resolve this issue (Mkenda et al., 2016).

Refreshments were important and arriving hungry may have reduced concentration and interaction. In Hong Kong, sessions involving food were most well received, as many participants’ historical backgrounds involved experiences of war and famine, therefore basic physiological needs held increased meaning (Wong et al., 2017).

### Strengths and limitations

Strengths of this study include the sample size which provides a rich data set, and the exploration of facilitators’ experiences alongside PwD. Consensus from multiple researchers improved the reliability and trustworthiness of the data. One limitation was that some group members had no formal dementia diagnosis. Although not interviewed, or included in this sample, this could have contributed to the group challenges. The potential impact is limited, as the screening process indicated a degree of cognitive impairment. Another limitation was the issue of asking PwD to remember experiences in detail. This could explain why participants shared more general emotional memories over specific examples.

Measures were taken to ensure correct translation of transcripts. Interviews were conducted by a non-native (JM) through an interpreter (ES), creating potential for misinterpretation. Linguistic nuances and subtle cues may have gone undetected. To limit this, a discussion was held after each interview to clarify intended meanings behind questions and answers.

### Implications for future research

Future research may involve a full RCT on CST in Tanzania. More broadly, the development of a questionnaire exploring the relationship between group processes and patient outcomes could then direct facilitators towards specific processes which might be enhanced or reduced to optimize treatment efficacy.

### Implications for future practice

Future implementation should consider grouping people of the same religion/tribe, achieving a gender balance, running groups in the mornings and providing refreshments. Finally, locals with understanding of cultural nuances and dialects could be trained as facilitators. This may improve acceptance and expectations of CST and implementation strategies.

## Conclusions

Exploration of participants’ and facilitators’ experiences of CST in Tanzania helped to identify several group processes. Overall, the group format offered many inter-connecting, positive experiences, supporting CST principles and corroborating improvements from quantitative research. Facilitators played an important role in eliciting specific group processes, as did the structure of sessions and range of activities specific to CST. Challenging group experiences often arose from cultural sources specific to rural Tanzania. Findings from this study offer deeper insight into individual experiences of CST and highlight areas for further development and optimization of treatment efficacy. They also support previous qualitative research into group mechanisms occurring within CST by offering participant and facilitator perspectives from a new cultural context.

## Appendix 1. Interview topic guide: Group members

Interview Topic Guide (for group members)

**Table:**

Case Number:	Location of interview:	Village:
**Date CST ended:**	**Date of Interview:**	**Number of people in CST group:**
**Religion:**	**Tribe:**	**Educational level:**

*Overall topic guide should be as exploratory as possible, therefore start with the ‘broad’ questions identified below. However, take not of the ‘more specific prompts’ within each section, which are specifically relevant for CST groups.*

### Topic 1: Generally exploring experiences of group processes

*Broad question:* **How did you feel being in the group of people?**

- Good / Positive experience? Bad / Negative experience? What way? How?

*More specific prompts:* **Did you mix/interact with others during the group?**

- Yes/No? Why not? How? Examples? When?
- Did you get to know others? Make friends?
- Were you helped by others? Did you trust others?
- Did you feel similar to others? How was talking to other people with dementia? How did it compare to day to day interactions?
- Did you feel involved or included?
- What did you talk about? Feelings/life problems?
- **If no, why not?**
- **Did you have the chance to talk in the group?**
- Why not?
- If yes- how easy was it?
- Hard to say what you wanted to others?
- Did you ever feel afraid to share your feelings with others?
- **What sort of things do you do with other people when you are not at the sessions?**
  - Has this changed since coming to the sessions?

### Topic 2: Highlighting CST principles: Exploring whether group processes had an impact on these?

*Broad question:* **Can you tell me about the activities you did in the group?**

Did being in a group with others affect your experience of these activities?

- Examples? Specific sessions you can describe this (how you would interact)?

*More specific prompts (if needed)*

- Did you feel mentally stimulated (active, engaged) – for example during sessions on food; childhood memories; music; number/word games?
- Did you have chance to discuss new things – for example during the sessions on faces session; current affairs sessions; opinions rather than facts?
- Helped to remember things / reminiscence - for example remembering past memories?
- Respect; involvement; inclusion; choice – for example during any creative activities; Food, maps

#### 3) Highlighting CST outcomes (cognition / quality of life): Exploring whether group processes had an impact on these?

##### From when you joined the group, has anything changed for you?

- Was the group ‘mentally stimulating’ (cognitive impact)?
  - Did the sessions help your memory?
  - What? Why? How?
- Do you feel any different overall since the sessions (quality of life)?
  - Feel any different overall? General well-being?
  - What? Why? How?
- If no: why not?

## Appendix 2 Interview topic guide: Group facilitators

**Interview Topic Guide (for group facilitators)**

*Overall topic guide should be as exploratory as possible, therefore start with the ‘broad’ questions identified below. However, take not of the ‘more specific prompts’ within each section, which are specifically relevant for CST groups.*

### Topic 1: Generally exploring experiences of group processes

Broad question: What was your experience of the group you facilitated?

- Good / Positive experience? Bad / Negative experience? What way? How?

More specific prompts: Did you feel that group members mixed/interacted with each other during the group?

- Yes/No? Why not? How? Examples? When?
- Did group members get to know each other? Make friends?
- Did group members help each other? Trust each other?
- Did the group members report/demonstrate feeling similar to each other?
- Did group members report/demonstrated being involved/included?
- Were group members of different religious backgrounds/tribes?
  - If so, did this cause any issues?
- **If not, why not?**

**What did group members talk about?**

- Feelings/life problems?
- **Did they have the chance to talk to others?**
- Hard to say what they wanted with others?
- Did they ever feel afraid to talk about problems?
- Was there an overall **sense of engagement**?
  - Did members try to understand why they did the activities?
  - Was there a sense of participation?
  - Did members challenge each other in their efforts to undertake group activities?
- Was there an overall **sense of avoidance**?
  - Avoidance of important issues?
  - Did group members depend on the group leader for direction?
  - Did group members appear to do things in the way they thought would be acceptable to the group?
- Was there an overall **sense of conflict**?
  - Friction/anger between members? Distance? Withdrawal? Evidence of rejection/distrust between group members? Did members appear to be tense/anxious?
- **Did group members report/demonstrate that the group had an impact on their social life outside the groups?**

### Topic 2: Highlighting CST principles: Exploring whether group processes had an impact on these?

Broad question: Can you tell me about the activities that happened in the group

#### Did group members participate in these activities together? If so, was there an impact of group members participating in these activities with each other?

- Examples? Specific sessions you can describe this (how you did group members interact)?

*More specific prompts (if needed)*

- Did group members report feeling mentally stimulated (active, engaged) – for example during sessions on food; childhood memories; music; number/word games?
- Did you they have chance to discuss new things – for example during the sessions on faces session; current affairs sessions; opinions rather than facts?
- Were group members helped to remember things / reminiscence - for example remembering past memories?
- Were group members respected/respectful; involved; included; given choice – for example during any creative activities; food, maps?

#### 3) Highlighting CST outcomes (cognition / quality of life): exploring whether group processes had an impact on these?

**Did you notice any changes in group members by the end of treatment; if so, were any of these changes impacted by the group format of the intervention?**

- Was the group ‘mentally stimulating’ (cognitive impact)?
  - Did being in a group help with memory?
  - What? Why? How?
- Did being in a group help members feeling a bit better about things (quality of life)?
  - Did group members report feeling any different overall? General well-being?
  - What? Why? How
- No: why not?
